# Fine mapping QTL for female fertility on BTA04 and BTA13 in dairy cattle using HD SNP and sequence data

**DOI:** 10.1186/1471-2164-15-790

**Published:** 2014-09-13

**Authors:** Johanna K Höglund, Goutam Sahana, Rasmus Froberg Brøndum, Bernt Guldbrandtsen, Bart Buitenhuis, Mogens S Lund

**Affiliations:** Department of Molecular Biology and Genetics, Center for Quantitative Genetics and Genomics, Aarhus University, P.O. Box 50, DK-8830 Tjele, Denmark; Department of Animal Breeding and Genetics, Swedish University of Agricultural Sciences, P.O. Box 7070, 750 07 Uppsala, Sweden

**Keywords:** Female fertility, Dairy cattle, Sequence analysis, Association study

## Abstract

**Background:**

Female fertility is important for the maintenance of the production in a dairy cattle herd. Two QTL regions on BTA04 and on BTA13 previously detected in Nordic Holstein (NH) and validated in the Danish Jersey (DJ) and Nordic Red (NR) were investigated further in the present study to further refine the QTL locations. Refined QTL regions were imputed to the full sequence data. The genes in the regions were then studied to ascertain their possible effect on fertility traits.

**Results:**

BTA04 was screened for number of inseminations (AIS), 56-day non-return rate (NRR), days from first to last insemination (IFL), and the interval from calving to first insemination (ICF) in the range of 38,257,758 to 40,890,784 bp, whereas BTA13 was screened for ICF only in the range from 21,236,959 to 46,150,079 with the HD bovine SNP array for NH, DJ and NR. No markers in the DJ and NR breeds reached significance. By analyzing imputed sequence data the QTL position on BTA04 was narrowed down to two regions in the NH. In these two regions a total of 9 genes were identified. BTA13 was analyzed using sequence data for the NH breed. The highest –log_10_(P-value) was 19.41 at 33,903,159 bp. Two regions were identified: Region 1: 33,900,143-33,908,994 bp and Region 2: 34,051,815-34,056,728 bp. SNPs within and between these two regions were annotated as intergenic.

**Conclusion:**

Screening BTA04 and BTA13 for female fertility traits in NH, NR and DJ suggested that the QTL for female fertility were specific for NH. A missense mutation in CD36 showed the strongest association with fertility traits on BTA04. The annotated SNPs on BTA13 were all intergenic variants. It is possible that BTA13 at this stage is poorly annotated such that the associated polymorphisms are located in as-yet undiscovered genes. Fertility traits are complex traits as many different biological and physiological factors determine whether a cow is fertile. Therefore it is not expected that there is a simple explanation with an obvious candidate gene but it is more likely a network of genes and intragenic variants that explain the variation of these traits.

**Electronic supplementary material:**

The online version of this article (doi:10.1186/1471-2164-15-790) contains supplementary material, which is available to authorized users.

## Background

The cow´s ability to reproduce is essential for milk production. Impaired reproduction will result in additional inseminations, higher replacement rate and increased culling rate. Recently genome sequencing technologies and bioinformatics analysis approaches have advanced tremendously. The availability of full genome sequence data can help to identify causal mutations underlying variation in female fertility. Application of these tools has led to remarkable increases in the numbers of trait markers available and thus enhanced precision of QTL mapping.

Genome-wide association studies (GWAS) examine common genetic variants in large numbers of individuals to determine whether an association with quantitative traits exists. GWAS have identified thousands of single nucleotide polymorphisms (SNPs) across the cattle genome associated with economically important traits in cattle (e.g. [1]). However, most of the genetic variants detected by these studies are not causal for the traits themselves. Instead they are in linkage disequilibrium (LD) with the causal polymorphisms. Most GWAS studies in cattle have used data from one breed only. In most cases this has been the Holstein Friesian (HF) where the largest datasets are available. The low effective population size in HF has resulted in long-range LD [2, 3]. This limits the studies’ ability to distinguish between causal factors and markers in strong LD with causal factors. The limitation can sometimes be overcome by using data from multiple breeds. A second limitation of previous mapping studies is that the marker panels used only represent a small fraction of the variants segregating in the population. Using a panel of individuals with whole genome sequence, in principle all SNPs can be imputed for all individuals in the mapping population [4]. The data thereby comes to include the causal variants. Causal effects thereby become identifiable.

In addition to revealing the genetic architecture that underlies the physiological and biological process of female reproduction, this information could also be practically applied to genomic selection schemes. Genomic prediction helps to select breeding animals for the next generation more efficiently. Introducing high density SNP arrays (777 k) did not substantial increase accuracy of genomic predictions in cattle (0.5 to 1%) as compared to medium density 50 k SNP arrays [5]. One of the reasons discussed was the increase in the number of unknown parameters to be estimated with high density data. With the availability of full genome sequence data this problem increases many folds. However, if the causal mutations underlying female fertility are identified and not only markers in LD with the causal mutation, the information could be included in genomic prediction models where additional weight can be put on certain genomic regions/variants which influence female fertility. This would in particular improve predictions over generations and across breeds.

Previously QTL areas influencing female fertility traits have been identified in the Nordic Holstein cattle population using the 50 k SNP array [6]. In this study two of these QTL regions previously identified [6] (BTA04 and BTA13) were first analyzed using the HD 777 k SNP array to further refine the regions in the NH. In addition, the Danish Jersey (DJ) and Nordic Red (NR) breeds were screened with the bovine HD array to investigate if the QTL on BTA04 and BTA13 were segregating in these breeds as well. To further refine the QTL regions in the NH, these regions were re-analyzed with the imputed full sequence data.

## Results

### HD SNP analysis

**Figure 1:**
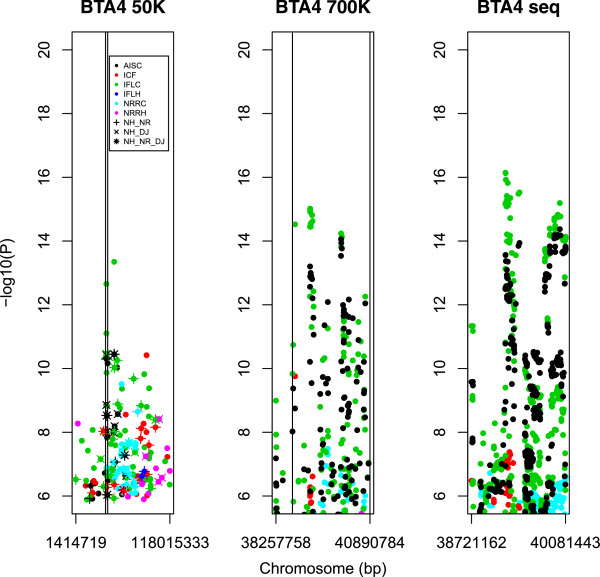
**Manhattan plots of GWAS on BTA04 for 5 female fertility traits with three densities of markers across successively narrower regions.** The y axes show –log_10_(p-value) of single-marker associations tests. The x axes show marker positions in base pairs (bp). Left panel: tests across three breeds with BovineSNP50 BeadChip [6]. Vertical lines indicate the region shown in the middle panel. Center panel: tests in Holstein with the HD BeadChip. Vertical lines indicate the region shown in the right panel. Right panel: tests using sequence data for the Nordic Holstein breed.

**Figure 2:**
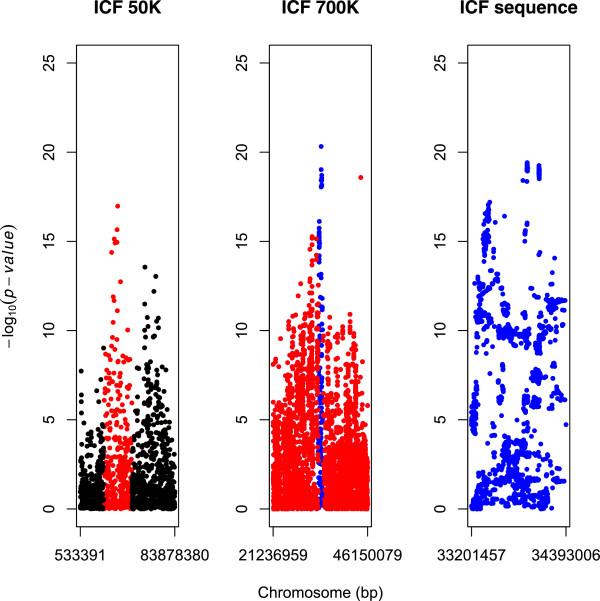
**Manhattan plots of the GWAS results on BTA13 for ICF with three densities of markers across successively narrower regions.** The y axes show –log_10_(p-value) of single-marker associations tests. The x axes show marker positions in base pairs (bp). Left panel: GWAS result of the Nordic Holstein breed with the BovineSNP50 BeadChip [6]. The red dots indicate the region shown in the center panel. Center panel: test result with the HD BeadChip. The blue dots indicate the region shown in the right panel. Right panel: test result using sequence data.

BTA04 and BTA13 were screened with the bovine HD SNP array in the NH, NR and DJ breeds, based on the positions on the genome which were chosen due to high significance from Höglund et al. [6]. BTA04 was screened for NRRH, IFLH, NRRC, ICF, IFLC, AISH, AISC in a 2,633,026 bp region spanning positions 38,257,758 to 40,890,784 bp (Figure 1: left), whereas BTA13 was screened for ICF only, in the region ranging from 21,236,959 to 46,150,079 bp (Figure 2: left). ICF was the only trait which showed significance in this position on BTA13 in the previous study [6]. Correlations of the minor allele frequencies of the most significant markers in the NH, DJ and NR pointed in the direction that the QTL for ICF is breed specific on BTA13. However the results of correlations between the minor allele frequencies of the most significant marker in the three breeds on BTA04 were inconclusive whether the QTLs were breed specific. SNP markers in the NH breed reached significance levels on both BTA04 and BTA13 (Figure 1: center and Figure 2 center; Additional files 1 and 2). However, no markers in the DJ and NR breeds reached significance analyzing HD SNP data.

### Sequence analysis

#### BTA04

Based on the results using the HD array (Figure 1: middle) an area from 38,721,162 – 40,890,784 bp was selected for further analysis with full sequence data for this region. Based on the sequence data analysis (Figure 1: right; Additional file 3) the QTL position was narrowed down to two regions. Region 1 spanned positions between 39,210,643 – 39,498,957 bp (=288,314 bp) and Region 2 spanned 39,700,194 – 40,890,784 bp (=1,190,590 bp). The two regions were searched for candidate genes/polymorphisms (Table 1). In total 9 genes were identified in these regions of which 5 were annotated, three were described as uncharacterized proteins and one was not annotated (Table 1). The annotated markers showing association with AISC, IFLC, NRRC and ICF are shown in Table 2. The annotated markers were not the markers with the highest –log_10_(P-value). The highest –log_10_(P-values) were 15.22 for AISC (Chr4:40298743), 16.14 for IFLC (Chr4:39213491), 7.18 for NRRC (Chr4:40728978) and 5.39 for ICF (Chr4:40599222).

**Table 1:** **Genes located on BTA04 in region 2 (39,700,194- 40,890,784 bp)**

Ensembl gene ID	Description	Gene start (bp)	Gene end (bp)	Gene name
ENSBTAG00000014800	Known pseudogene	40067664	40068936	
ENSBTAG00000006138	Semaphorin-3C	40140494	40345588	SEMA3C
ENSBTAG00000047646	Uncharacterized protein	40432906	40457597	
ENSBTAG00000014220	Uncharacterized protein	40457450	40560780	
ENSBTAG00000046905	Uncharacterized protein	40541701	40542459	
ENSBTAG00000017866	Platelet glycoprotein 4	40581484	40643369	CD36
ENSBTAG00000008641	guanine nucleotide-binding protein G(t) subunit alpha-3	40775885	40830619	GNAT3
ENSBTAG00000029292	5S ribosomal RNA	40887316	40887433	5S rRNA
ENSBTAG00000044967	U6 spliceosomal RNA	39753149	39753244	U6

**Table 2:** **Annotation of the markers on BTA04 associated with AISC, IFLC, NRRC and ICF in region 2 (39,700,194- 40,890,784 bp)**

Marker	Description	Position (bp)	AISC ^1^ -log(p-value)	IFLC ^1^ -log(p-value)	NRRC ^1^ -log(p-value)	ICF ^1^ -log(p-value)	Gene name
rs43384664	Non coding exon variant	39753216	2.4605	2.8191	0.8507	0.7718	
rs43383647	Non coding exon variant	40067725	2.5025	2.5839	1.8574	0.2769	
rs43383646	Non coding exon variant	40067763	4.3396	3.4117	2.4495	0.7627	
rs43383645	Non coding exon variant	40067774	2.5029	2.5845	1.8577	0.2770	
rs43383644	Non coding exon variant	40067834	2.5273	2.6006	1.8879	0.2715	
rs43383643	Non coding exon variant	40067978	3.9303	5.1706	1.8290	1.7758	
rs109658404	Non coding exon variant	40068059	2.0745	2.0725	1.3027	0.2634	
rs43383637	Non coding exon variant	40068666	3.9227	5.1547	1.8272	1.7656	
rs43383636	Non coding exon variant	40068699	3.9227	5.1547	1.8272	1.7656	
rs109959240	Non coding exon variant	40068766	2.1516	2.1473	1.3416	0.2780	
rs136871729	Non coding exon variant	40068903	2.1559	2.1552	1.345	0.2807	
40322885C/A	Synonymous variant	40322885	2.7487	4.1129	0.2773	4.5324	SEMA3C
40438940A/G	Missense variant	40438940	1.4299	1.2607	0.4097	0.3905	UP
rs136410227	Synonymous variant	40455017	6.7135	7.3694	2.8445	2.6552	
40585702G/A	Synonymous variant	40585702	12.8399	14.0147	5.3021	5.3908	CD36
40599222G/A	Synonymous variant	40599222	13.1476	14.2355	5.4904	5.3956	CD36
40614608C/T	Missense variant	40614608	13.2259	14.2777	5.5281	5.3700	CD36
40614675G/A	5 prime UTR variant	40614675	13.2255	14.2828	5.5249	5.3764	CD36
40807452A/G	Missense variant	40807452	9.2722	8.5549	4.1428	2.0438	GNAT3
rs110078696	Synonymous variant	40807576	9.358	8.6351	4.2066	2.0567	GNAT3
rs109903966	Synonymous variant	40807594	9.3572	8.6346	4.2061	2.0566	GNAT3
rs108969608	Synonymous variant	40813977	9.4276	8.7599	4.2175	2.1207	GNAT3

^1^AISC: Number of inseminations per conception; IFLC: days from first to last insemination; NRRC: 56-day non-return rate; ICF: the length in days of the interval from calving to first insemination.

#### BTA13

**Figure 3:**
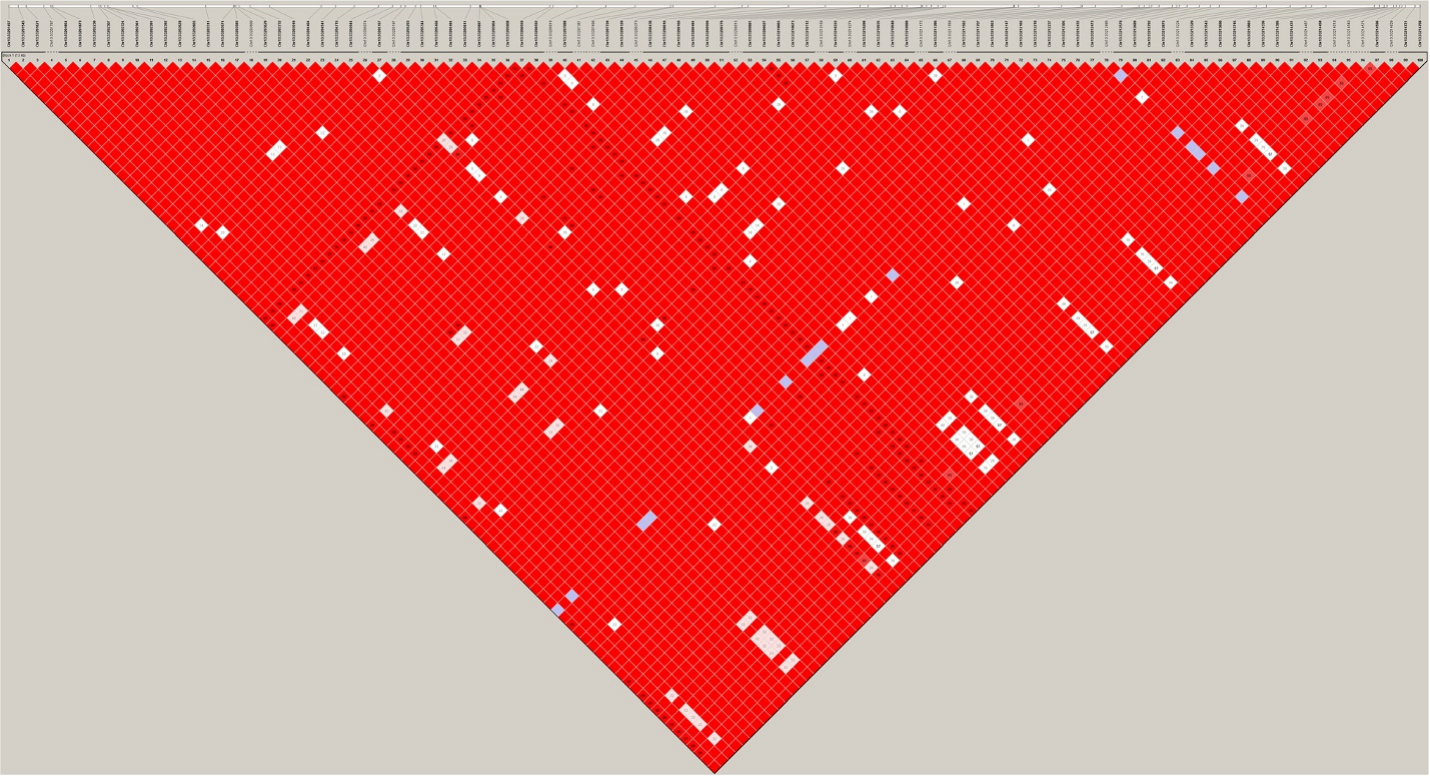
**Linkage disequilibrium plot of the pairwise D’ for the identified regions for the length in days of the interval from calving to first insemination (ICF) on BTA13.**

Based on the results of the HD array (Figure 2: middle) an area from 33,201,457 bp to 34,393,006 bp was analyzed using sequence data (Additional file 4). The highest –log_10_(P-value) was 19.41 at 33,903,159 bp. Based on the analysis using sequence data two regions were identified (Figure 2: right): Region 1: 33,900,143-33,908,994 bp (=8,851 bp) and Region 2: 34,051,815-34,056,728 bp (=4,913 bp). Linkage disequilibrium (LD) analysis showed that these two regions are in almost complete LD (Figure 3). BTA13 was screened for candidate genes/polymorphisms in the range of 33 Mb to 34.4 Mb. In total 8 genes were identified in the area from 33.2 Mb to 34.4 Mb in of which 6 were annotated, one was described as uncharacterized protein and one was described as a pseudogene (Table 3). These genes were not located in Region 1 and Region 2 (Additional file 5: Figure S1). The SNPs in Region 1 and Region 2 as well as the region in-between were annotated by Variant Effect Predictor as “intergenic” i.e. no candidate genes were identified.

**Table 3:** **Genes located on BTA13 in the range of 33,000,000 bp to 34,400,000 bp**

Ensembl gene ID	Description	Gene start (bp)	Gene end (bp)	Gene name
ENSBTAG00000022000	calcium channel, voltage-dependent, beta 2 subunit	33005028	33285264	CACNB2
	uncharacterized LOC100847770	33281429	33283899	LOC100847770
ENSBTAG00000022003	NOP2/Sun domain family, member 6	33291604	33369610	NSUN6
ENSBTAG00000001552	enhancer of polycomb homolog 1 (Drosophila)	33451679	33518736	EPC1
ENSBTAG00000002280	kinesin family member 5B	33619259	33665129	KIF5B
ENSBTAG00000012677	Rho GTPase activating protein 12	33713676	33830942	ARHGAP12
ENSBTAG00000020053	zinc finger E-box binding homeobox 1	34061282	34261303	ZEB1
	deoxyuridine triphosphatase pseudogene	34103073	34103547	LOC100141025

## Discussion

By analyzing the results of the significant QTL presented earlier [6] using the HD data, it became evident that BTA04 and BTA13 showed the most significant results.

In this study we have re-analyzed two regions which were previously associated to female fertility traits and validated in different breeds of dairy cattle [6]. A single marker analysis was performed using the HD array and subsequently DNA sequence for BTA04 and BTA13, respectively. The physical location of the SNPs has been used to search for candidate genes on the bovine genome.

### BTA04

In the QTL region 9 genes were annotated (Table 1). Based on the –log_10_(P-value) (Table 2), CD36 is the most obvious candidate gene. CD36 has a missense variant. A missense variant is a sequence variant that changes one or more bases, resulting in a different amino acid sequence but where the length is preserved (http://www.sequenceontology.org/miso/current_release/term/SO:0001583). CD36 has been suggested to be involved in numerous potential physiological functions; cell adhesion processes, binds long chain fatty acids and may function in the transport and/or as a regulator of fatty acid transport and act as receptor for thombospondins, THBS1 AND THBS2, mediating their antiangiogenic effects. This in turn involves many physiological processes [7].

Another interesting candidate on BTA04 based on its suggested biological function is Semaphorin-3C (SEMA3C). SEMA3C binds to members of the plexin family and plays an important role in the regulation of developmental processes. Furthermore it is required for normal cardiovascular development during embryogenesis and plays an important role in axon growth and axon guidance by functioning as attractant for growing axons (http://www.uniprot.org/uniprot/A7MB70). However, the SNP markers located in these genes did not reach genome wide significance for the traits AISC, IFLC, and NRRC (Table 2). At this stage annotation of this region on BTA04 is not sufficient to reach a firm conclusion about the causal polymorphism. Even though some of the SNP markers were located within genes, the SNP markers with the most significant associations to AISC and IFLC were not annotated. Therefore, a better annotation might help to identify the nature of the causal mutation.

### BTA13

The HD analysis revealed a sharp peak on BTA13 in the range of 33.2 Mb to 34.4 Mb. Searching the chromosome for genes revealed a low number of annotated genes (918 transcripts on the entire chromosome). Eight of these genes were in the 33.2 Mb to 34.4 Mb range. The sequence analysis revealed two regions within the 33.2 Mb to 34.4 Mb range. Even though we have used sequence data it is still a challenge to disentangle whether the peaks harbor the same genetic variation influencing ICF, or are in reality a combination of two separate genetic variations. Therefor the two peaks were analyzed further and were determined to be in high LD. This suggests that it is the same genetic variation causing the two peaks. The SNPs annotated in these two regions were all annotated as intergenic variants. It is possible that BTA13 at this stage is poorly annotated such that the associated polymorphisms are located in as-yet undiscovered genes.

Fertility traits are complex traits as many different biological and physiological factors determine whether a cow is fertile, another challenge is the way the phenotypes are defined, they do not distinguish between different biological functions as they are defined more for a breeding purpose than from a biological point of view. Therefore it is not expected that there is a simple explanation with an obvious candidate gene, it is more likely a network of genes and intragenic variants that explain the variation of these traits. A number of genes and their potential functions in the vicinity of the two peaks are presented in Table 3.

### Selection of markers

The two regions on BTA4 and BTA13 were chosen based on previous studies by the authors [6, 8]. The SNP markers for BTA04 and BTA13 from the bovine HD array have been tested in the three breeds. Even though the number of sires in the NR and DJ breeds are much lower compared to the NH breed the results suggest that the QTL for female fertility traits detected on both chromosomes are specific for the NH breed. What appeared to be the same hits on several chromosomes in a previous manuscript in several breeds when using the 50 k SNP array, clearly was not when using the 700 k SNP array.

We have been able to narrow down the regions on BTA04 and BTA13 by adding many more markers. However in our analysis we could not with certainty pinpoint the causal polymorphism for the fertility QTL. This might be due to the fact that half of the total genetic variants identified in the whole genome sequencing (WGS) were filtered out for various reasons. All the variants which were not bi-allelic were dropped due to limitations in the imputation software. Therefore, the actual causative polymorphism may be missing from the data analyzed here. Furthermore, there were hundreds of SNPs with very high –log_10_(P-values) due to high LD among themselves. Therefore, it is not possible to pick a few from them based on this analysis.

### Effect of recessive lethals on fertility in Dairy Cattle

Recently it has been argued that an increase in recessive lethals play a part in the decline of fertility as elite sires obtain a very large number of offspring in cattle breeding, and the effective population size is around 50 in the Holstein breed [9]. The development of increased information on the cattle genome has enabled us to detect putative recessive lethal mutations by the absence (or near absence) of homozygous recessive individuals in the population [10]. Some recessive lethal alleles are known to affect the developing embryo. A recent study in the Nordic Holstein population [10] has identified a number of recessive lethal haplotypes which appear to act in early pregnancy. The carrier frequency of the recessive lethal alleles is up to 20% in the Nordic Holstein population. Also, VanRaden et al. [11] and Fritz et al. [12] have mapped recessive lethals genes by homozygosity mapping in Holstein populations. However in the present study it does not seem to be the same effects that are picked up as the recessive lethals are not located on BTA04 or BTA13.

### Annotation of the bovine genome

Tools and resources for annotation and gene discovery in the bovine genome are available (e.g. http://www.ncbi.nlm.nih.gov/assembly/GCF_000003055.4/; [1]). Even though many genes have already been annotated on the bovine genome (http://www.ncbi.nlm.nih.gov/assembly/GCF_000003055.4/), the known set sites of gene transcription, initiation, termination as well as differential splicing remains incomplete. Therefore, information on genomic structure of organisms which are better annotated like mouse and human, are used as an information source to cover these gaps in the knowledge of the genome structure. However, identifying regulatory elements and non-protein coding regions annotation remains more challenging [8, 9].

### Regulatory elements in the genome

It is a challenge to identify all regulatory elements in the genome also those which control gene expression. The question is how to relate genes and their products function. Even though we have access to the cattle sequence which facilitates the comprehensive identification of these transcriptional regulatory factors there is still a long way to go. The expressions of eukaryotic protein coding genes are regulated in different steps which include elements like; transcription initiation and elongation, mRNA processing, transport, translation and stability. Most regulation is believed to occur at the level of transcription [13].

Directly measured phenotypes and genotypes of cows are necessary to provide a more direct link between phenotype and genotype, it can also enable mapping of non-additive effects which affect fertility traits in cattle [14]. This was not investigated in the present study but could potentially shed more information regarding fertility in cattle.

Identification and analysis of phenotypic measures that reflect more directly the physiologic background of the reproduction traits could also be helpful in determining the precise physiological background represented by a specific QTL. Analyzing expression data from reproductive organs might help to identify the temporal-specific aspects of gene expression.

## Conclusions

The results from screening BTA04 and BTA13 of NH, NR and DJ using the bovine HD SNP array for female fertility traits suggested that the QTL for female fertility located on these chromosomes were specific for the NH breed. The subsequent screening using the imputed sequence variants for NH narrowed down the QTL on BTA04 into two regions of 288,314 bp and 1.1 Mb respectively, while on BTA13 the QTL region for ICF was narrowed down into two regions of 8,851 bp and 4,913 bp, respectively. A candidate gene search for these QTL regions revealed that a missense mutation in the Platelet glycoprotein 4 (CD36) gene showed the strongest association with fertility traits and therefore is a strong candidate for the QTL on BTA04, whereas the annotated SNPs on BTA13 were all intergenic variants. Fertility traits are complex traits as many different biological and physiological factors determine whether a cow is fertile. Therefore it is not expected that there is a simple explanation with an obvious candidate gene but it is more likely a network of genes and intragenic variants that explain the variation of these traits.

## Methods

### Animal ethics statement

Phenotypic data were obtained from routine records of cattle farms. Semen and blood samples for genotyping were collected in previous studies [15, 16] using standard procedure for breeding purposes by veterinary or authorized personnel, and all efforts were made to minimize suffering.

No data was collected for the purposes of this study. All DNA data were obtained through the analysis of materials collected as part of routine operation of cattle breeding programs. Farm management and breeding programs in the EU and EEA are subject to the “European Convention for the Protection of Animals kept for Farming Purposes” as implemented in national law.

### Animal population

The animal population has been described before in Höglund et al. [6]. In short, a total of 3,475 Nordic Holstein (NH) sires from Denmark, Sweden and Finland with official breeding values for female fertility traits were used to discover associations. We used 4,998 Nordic Red (NR) animals and 1,225 Danish Jersey (DJ) animals to check for segregation for QTLs in these populations.

### Phenotypes

The traits evaluated included: number of inseminations per conception (AIS), 56-day non-return rate (NRR), days from first to last insemination (IFL), and the length in days of the interval from calving to first insemination (ICF). With exception of ICF, single trait breeding values (STBVs) from the national evaluation were available for both 1^st^ parity animals (heifers, suffixed H) and 2^nd^ and 3^rd^ parity animals (cows, suffixed C). For details regarding the phenotypes recorded and models used in routine breeding value prediction, see http://www.nordicebv.info.

### Sequencing

The sequences for the reference population used for imputation of Nordic animals consisted of the whole genome sequence carried out at Aarhus University and in the 1,000 Bull Genome project [4]. The sequencing of Nordic bulls at Aarhus University, Foulum was done using Illumina sequencers at Beijing Genomics Institute, Shenzhen, China. Sequencing was shotgun paired-end sequencing with a read length of 91 base pairs. Fastq data were converted from Illumina to Sanger quality encoding using a patched version of maq [17]. They were aligned to the UMD3.1 assembly of the cattle genome [18] using bwa version 0.6.2 [19]. They were converted to raw BAM files using samtools [20]. Quality scores were re-calibrated using the Genome Analysis Toolkit [21] version 1.6’s following the Human 1000 Genome guidelines incorporating information from dbSNP version 133 [22]. Sequences were realigned around insertion/deletions using the Genome Analysis Toolkit version 1.6. Variants were called using the Genome Analysis Toolkit version 1.6’s UnifiedGenotyper. Genomes for the 1,000 Bull Genomes project were sequenced in a number of laboratories and collected in the Department of Primary Industries, Victoria, Australia. Data processing was standardized. Sequences were aligned to the same reference genome as used at Aarhus University using versions of bwa [19]. Variant calling was done using samtools’s mpileup function. Variant Call Files from Aarhus University and the 1,000 Bull Genomes project were combined using the Genome Analysis Toolkit’s CombineVariants with precedence given to calls from the Nordic dataset for animals appearing in both datasets.

### Imputation HD and Sequence data

The sires used in this study were genotyped using the 50 k SNP array as described earlier [6]. These 50 k SNP typings were the basis for the imputation to HD and sequence level. The imputation of 50 k SNP to the full sequence was done in two steps. First in another study (N.K. Kadri, *pers. comm*.), the 50 k genotypes (46,702 SNPs after quality control) for 12,322 Nordic bulls were imputed to HD genotypes (734,077 SNPs) using the software IMPUTE2 [23]. The reference population with HD genotypes was available for 2,036 bulls (902 Holstein, 735 Nordic Red and 399 Danish Jersey).

In the second step of imputation, the 12,322 bulls imputed to HD genotypes were further imputed to the full sequence level, using a reference of 242 sequenced dairy bulls (132 Holstein, 42 Jersey, 52 Nordic Red and 16 Brown-Swiss). The 242 dairy cattle sequences originated from a combination of sequences processed at Aarhus University and sequences from the 1,000 Bull Genomes dataset. Only polymorphisms identified both in the Aarhus University dataset and the 1,000 Bull Genomes dataset were included. For positions containing both a SNP and an INDEL, the INDEL was deleted. SNPs at positions with disagreements between alleles from sequence and HD data were deleted. The reference data was pre-phased with BEAGLE v3.3.2 [24] and all markers with an r^2^ value below 0.9 were removed. This left a total of 16,374,063 sequence markers and 629,028 HD markers for chromosomes 1–29. Chromosomes were divided into chunks of about 20,000 consecutive sequence markers with an overlap of 500 markers to minimize imputation error at ends of the chunks. Imputation was done using BEAGLE v3.3.2 [24].

### Statistical method for association analysis

The association between the SNP and the phenotype was assessed by a single-locus regression analysis for each SNP separately, using a linear mixed model [25]. The model was as follows:


where **y** is the vector of phenotypes (de-regressed EBV), 1 is a vector of ones with length equal to the number of observations, *μ* is the general fixed mean, ***m*** is a vector of genotypic indicators (-1, 0, or 1) associating records to the marker effect, *g* is a scalar value of the additive fixed allele substitution effect of the SNP, ***Z*** is an incidence matrix relating phenotypes to the corresponding random polygenic effect, ***u*** is a vector of the random polygenic effect with a multivariate normal distribution N  where ***A*** is the additive relationship matrix and  is the polygenic variance, and *e* is a vector of random environmental deviates with a normal distribution ) where  is the error variance and ***W*** is the diagonal matrix containing weights of the de-regressed estimated breeding values. The weight for the i^th^ animal was estimated as *w*_*i*_ = *r*^2^/(1 - *r*^2^), where r^2^ was the reliability of the de-regressed EBV of the i^th^ animal and *r*^2^ > 0.98 was set to 0.98 to avoid very large weights for sires with very large number of progeny records. The model was fitted by REML using the software DMU [26]. The standard error of the fixed effect estimates was obtained from DMU. Testing for the presence of an effect of a marker was done using a Wald test against a null hypothesis of H_0_: g = 0.

### Significance levels

The significance level using genome wide bonferoni correction was 0.05/38,545 ~ 1.3 × 10^-6^ in the 50 k data (previous study). It was 0.05/734,077 ~ 6.8 × 10^-5^ SNPs in the 700 k data and 5.5 × 10^-8^ in the WGS data. The significance level using region wise bonferoni correction was 0.05/3716 ~ 1.3 × 10^-6^on BTA04 and on BTA13 0.05/3944 ~ 1.3 × 10^-4^.

### Variant annotation

Variants were annotated using Variant Effects Predictor version 2.8 [27]. The underlying databases correspond to ENSEMBL databases version 70.

### Linkage disequilibrium

To distinguish whether the two peaks in the sequence analysis on BTA13 were due to two separate mutations or one mutation the linkage disequilibrium of BTA13 was examined for these peaks. The top 100 markers in each peak were examined using HAPLOVIEW [28].

### Availability of supporting data

All DNA sequences used were taken from a publicly available assembly. The assembly is available for download (ftp://ftp.ensembl.org/pub/release-73/fasta/bos_taurus/dna). All variations used in the mapping study have been submitted by the 1000 Bull Genomes project for inclusion in dbSNP (http://www.ncbi.nlm.nih.gov/SNP). All annotations were obtained from a publicly available source (http://www.ensembl.org) downloadable including through Variant Effect Predictor (http://www.ensembl.org/info/docs/tools/vep/script/index.html).

Samples were collected in the context of previous studies [15, 16]. The pool of HD chip genotypes used as reference for imputations was created from a number of sources [29]. Contributions were obtained through exchange agreements with other research institutions in Sweden, Finland, Germany, France, The Netherlands and Spain. Other genotypings were done commercially on behalf of Aarhus University. Access to HD chip typing data can be granted by each exchange partner individually.

## Electronic supplementary material

Additional file 1: **Results of BTA04 in the Danish Holstein breed for number of inseminations per conception (AIS), 56-day non-return rate (NRR), days from first to last insemination (IFL), and the length in days of the interval from calving to first insemination (ICF) using the bovine HD SNP array.** (TXT 148 KB)

Additional file 2: **Results of BTA13 in the Danish Holstein breed for the length in days of the interval from calving to first insemination (ICF) using the bovine HD SNP array.** (TXT 242 KB)

Additional file 3: **Results of the single-marker analysis of BTA04 in the Danish Holstein breed for number of inseminations per conception (AIS), 56-day non-return rate (NRR), days from first to last insemination (IFL), and the length in days of the interval from calving to first insemination (ICF) using sequence data.** Only significant P values are mentioned for each trait. NA: marker not significant. (TXT 70 KB)

Additional file 4: **Results of the single-marker analysis of BTA13 in the Danish Holstein breed for the length in days of the interval from calving to first insemination (ICF) using sequence data.** Only region-wide significant P-values are presented. (TXT 91 KB)

Additional file 5: **Manhattan plots of the GWAS results using imputed DNA sequence on BTA13 for ICF in the range of 33.2 Mb to 34.4 Mb.** The y axes show –log_10_(p-value) of single-marker associations tests. The x axes show marker positions in base pairs (bp). The blue dots indicate test results using sequence data. The red triangles on the x-axis indicate the position of the annotated genes in the region. (PDF 26 KB)
