# Supplementary figures and images for: Fine mapping QTL for female fertility on BTA04 and BTA13 in dairy cattle using HD SNP and sequence data
Source: BMC Genomics. 2014 Sep 13;15(1):790. doi: 10.1186/1471-2164-15-790 (PMC4169824; doi:10.1186/1471-2164-15-790)

# ICF sequence

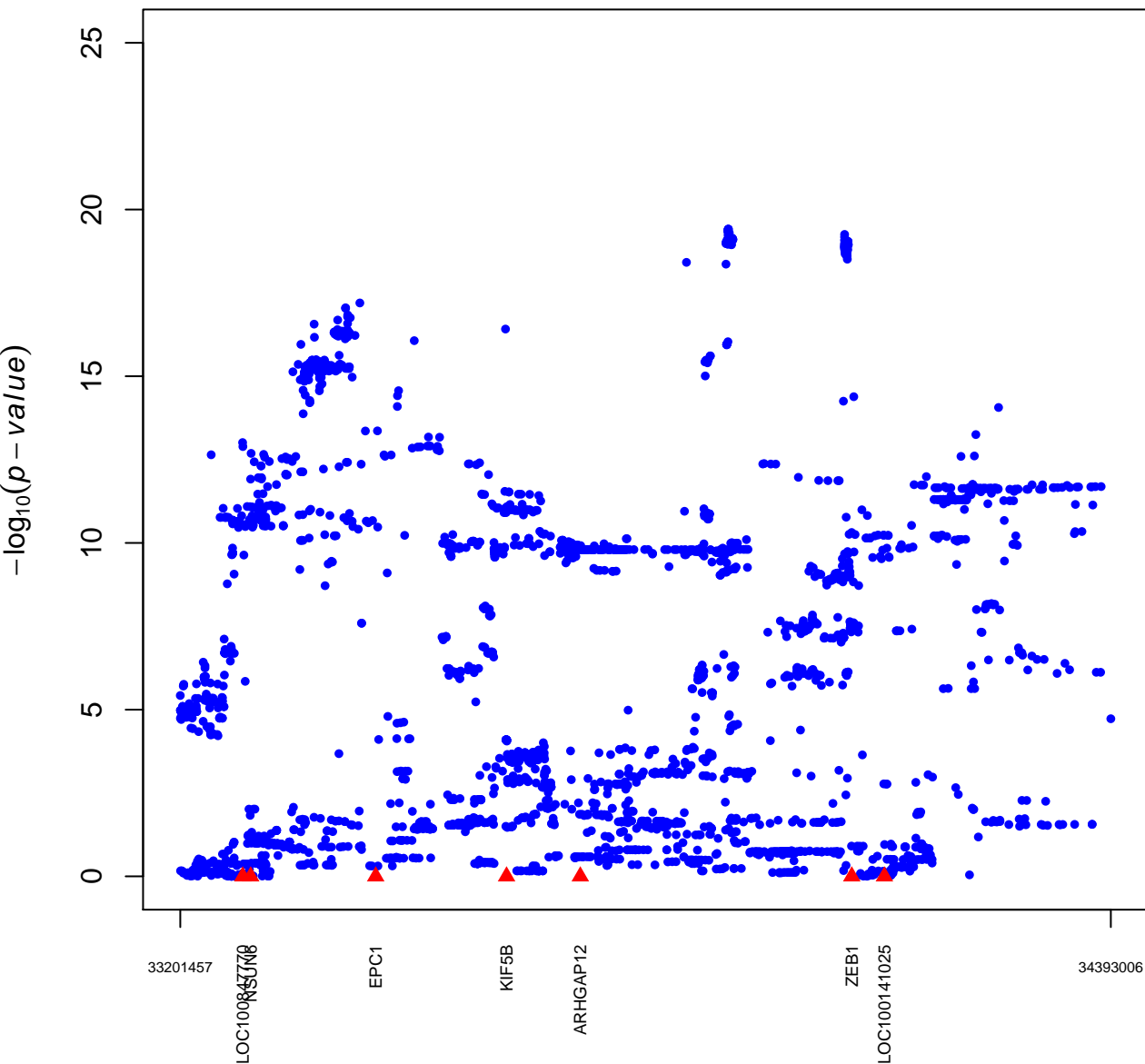

Supplement: Supplementary file 5 — Additional file 5: Manhattan plots of the GWAS results using imputed DNA sequence on BTA13 for ICF in the range of 33.2 Mb to 34.4 Mb. The y axes show –log10(p-value) of single-marker associations tests. The x axes show marker positions in base pairs (bp). The blue dots indicate test results using sequence data. The red triangles on the x-axis indicate the position of the annotated genes in the region. (PDF 26 KB) [file 12864_2013_6460_MOESM5_ESM.pdf]
